# Sex-specific impact of computed tomography–defined sarcopenia on postoperative complications following elective cardiac surgery in older patients

**DOI:** 10.1016/j.xjon.2026.101975

**Published:** 2026-07-27

**Authors:** Hitomi Ueda, Yoshiaki Kubota, Yuji Maruyama, Takuya Nishino, Kuniya Asai, Yosuke Ishii

**Affiliations:** a Department of Cardiovascular Surgery, Nippon Medical School, Tokyo, Japan; b Department of Cardiovascular Medicine, Nippon Medical School, Tokyo, Japan; c Department of Health Policy and Management, Nippon Medical School, Tokyo, Japan

**Keywords:** cardiac surgery, computed tomography, frailty, postoperative complications, sarcopenia, sex-based differences

## Abstract

**Objectives:**

Sex-specific differences in sarcopenia-related prognosis after cardiac surgery are unclear. We aimed to investigate the association between preoperative computed tomography–defined sarcopenia and postoperative complications in older patients undergoing elective cardiac surgeries, focusing on sex-specific differences.

**Methods:**

This retrospective observational study included 133 patients aged 65 years or more who underwent elective open surgeries. Sarcopenia was assessed using the psoas muscle area index derived from preoperative computed tomography scans and defined using sex-specific cutoff values. The primary outcome was postoperative complication.

**Results:**

Forty-nine patients (36.8%) had sarcopenia. Although the incidence of complications was higher in this group, the difference was not statistically significant between the groups. Multivariate logistic regression analysis excluding patients with preexisting atrial fibrillation revealed that sarcopenia was independently associated with a higher risk of postoperative complications (odds ratio, 3.73, 95% CI, 1.49-9.84, *P* = .006). The male patients with sarcopenia showed a higher incidence of complications versus those without (56.8% vs 33.9%, *P* = .046), whereas no association was observed in the female patients. Sex-stratified analyses confirmed the independent prognostic value of sarcopenia in male patients only (odds ratio, 2.52, 95% CI, 1.08-6.01, *P* = .033).

**Conclusions:**

Preoperative computed tomography–defined sarcopenia was independently associated with postoperative complications in older male patients undergoing elective cardiac surgeries, but not in female patients. These findings suggest that the prognostic significance of sarcopenia is sex-specific and highlight the importance of sex-specific risk stratification strategies in older patient populations undergoing cardiac surgeries.

## Graphical Abstract

**Figure:**
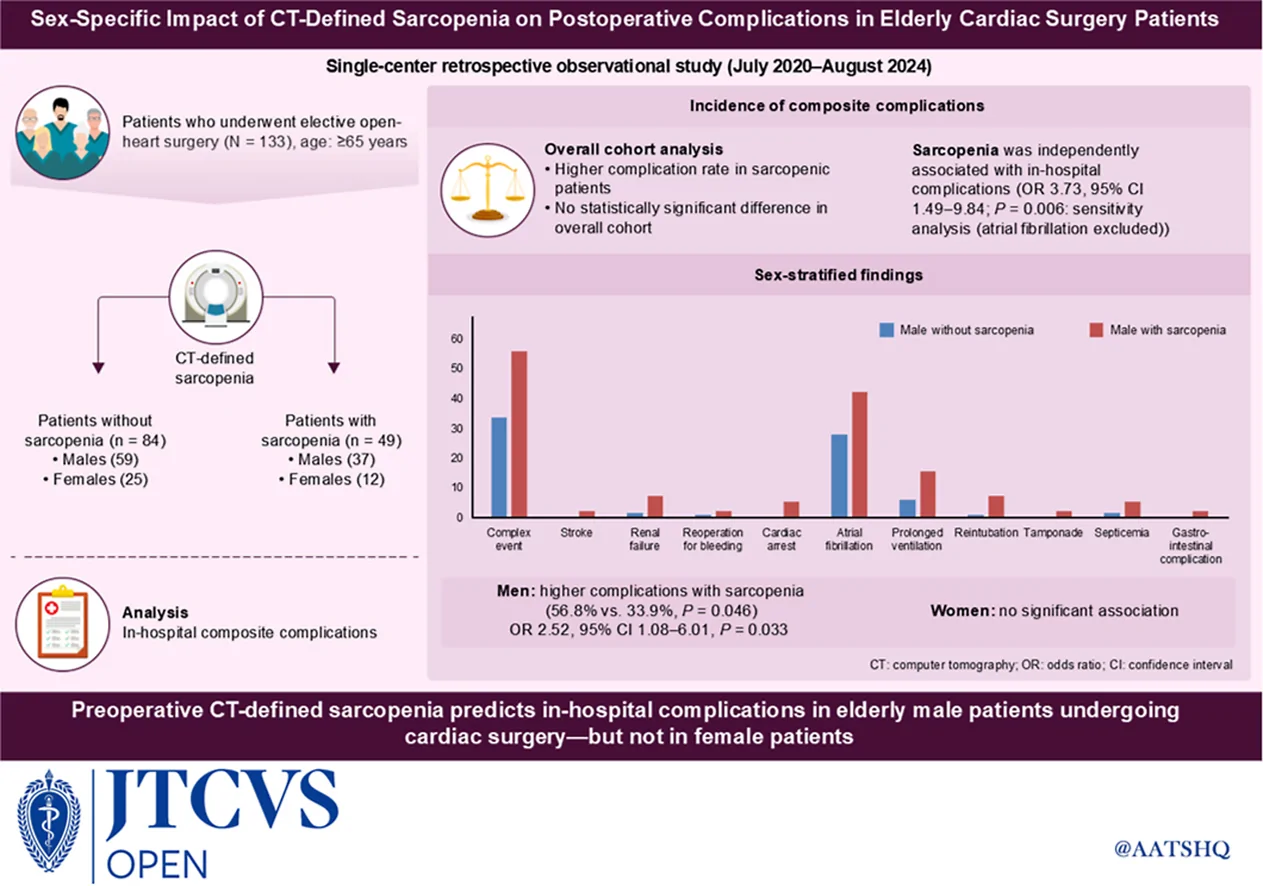

**Figure:**
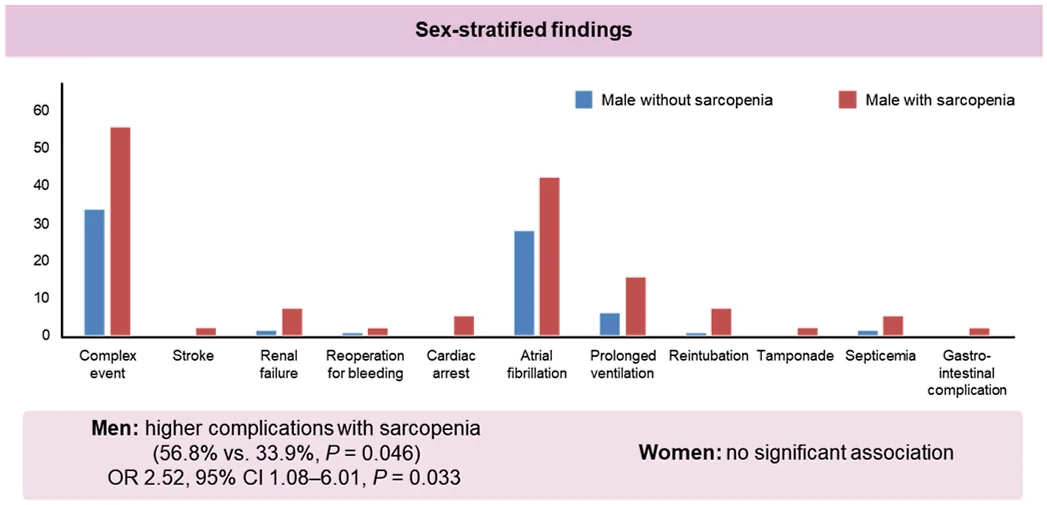
Preoperative CT-defined sarcopenia predicts in-hospital complications in older male patients undergoing cardiac surgery, but not in female patients.

**Central Message**

Preoperative sarcopenia in older adults undergoing elective cardiac surgeries was linked to worse postoperative outcomes in male patients with potential implications for surgical risk stratification.

**Perspective**

Researchers and practitioners in the field are likely to interact with older adults who may present with sarcopenia; in research and clinical practice, these patients may need to be screened or managed differently from the general population.

Cardiac surgery imposes substantial physiological stress on patients, particularly older adults with multiple comorbidities. Despite advances in surgical techniques and perioperative management, postoperative complications remain common after open surgeries and have been associated with prolonged hospitalization, increased healthcare resource use, and poor clinical outcomes.^1^, ^2^, ^3^ Therefore, accurate preoperative risk stratification is essential to identify vulnerable patients and optimize perioperative management strategies. Older patients undergoing cardiac surgeries are at particularly high risk for adverse postoperative outcomes.^4^, ^5^, ^6^ Age-related declines in physiological reserve, combined with comorbid conditions, contribute to a higher susceptibility to postoperative complications.^7^, ^8^, ^9^ Conventional risk assessment tools primarily focus on cardiac function and comorbidities; thus, they may not fully capture the impact of frailty-related factors on postoperative outcomes.

Sarcopenia, characterized by a reduction in skeletal muscle mass, has emerged as a key component of frailty that has been associated with higher morbidity and mortality in older populations.^10^, ^11^, ^12^, ^13^ In surgical settings, sarcopenia has been linked to higher rates of postoperative complications, prolonged hospital stays, and adverse outcomes across various surgical disciplines.^14^, ^15^, ^16^, ^17^, ^18^ Several studies have demonstrated the prognostic value of computed tomography (CT)-defined sarcopenia in terms of predicting postoperative outcomes.^16^, ^17^, ^18^, ^19^ However, evidence regarding its impact on postoperative complications in patients undergoing cardiac surgeries remains limited, and the potential influence of sex-specific differences in such sarcopenia assessments has not been fully elucidated.

Therefore, in this study, we aimed to investigate the association between preoperative CT-defined sarcopenia and postoperative complications in patients aged 65 years or more undergoing elective open surgeries. We also sought to explore sex-specific differences in the prognostic significance of sarcopenia, given the known differences in body composition and muscle mass distribution between male and female patients.

## Materials and Methods

### Study Design and Participants

This retrospective observational study included patients aged 65 years or more who underwent elective open surgeries at Nippon Medical School Hospital between July 2020 and August 2024. Among 472 consecutive patients who underwent open surgery during the study period, some were excluded if they underwent nonelective surgeries, were aged less than 65 years, or had insufficient clinical or imaging data for our analysis. After applying these exclusion criteria, the remaining 133 patients were included in the final study population.

The study was conducted in accordance with the principles of the Declaration of Helsinki. The study protocol was reviewed and approved by the Institutional Committee on Human Research at Nippon Medical School Hospital (Institutional Review Board Approval Number: B-2020-325, date of approval March 8, 2021). Informed consent was obtained using an opt-out approach, with study-related information provided via posters at the hospital. The opt-out information provided to patients stated that anonymized study data could be used for academic presentations and scientific publications.

### Sarcopenia Definition

Sarcopenia was assessed using preoperative CT images. The psoas muscle area was measured at the level of the third lumbar vertebra on axial CT images obtained during routine preoperative evaluations. The bilateral psoas muscle areas were manually traced and summed, and the psoas muscle area index (PAI) was calculated by dividing the total psoas muscle area by the body surface area (cm^2^/m^2^). CT images were analyzed by 2 independent physicians, and any discrepancies were resolved through consensus. Inter- and intra-rater reliability were consistently high. Sarcopenia was defined according to sex-specific PAI cutoff values, at less than 6.89 cm^2^/m^2^ for male patients and less than 4.4 cm^2^/m^2^ for female patients. The patients were classified into sarcopenia and nonsarcopenia groups based on these criteria.^20^

### Data Collection

The data extracted from the patients’ medical records included age, sex, diagnosis, comorbidities, treatment modalities (including medication history). Data concerning medication history, blood test results, and echocardiography findings were collected preoperatively. Left ventricular ejection fraction was calculated using the Teichholz or modified Simpson methods.

### Outcomes

The primary outcome was the occurrence of postoperative complications, defined according to the criteria of the Japanese Society for Cardiovascular Surgery. The composite end point consisted of any of the following complications during postoperative hospitalization: stroke, renal failure requiring renal replacement therapy, reoperation for bleeding, cardiac arrest, atrial fibrillation, prolonged mechanical ventilation, reintubation, tamponade, septicemia, or gastrointestinal complications. The incidence of the primary outcome was evaluated in the overall cohort and then further analyzed after stratification by sex. Postoperative atrial fibrillation events included both new-onset and recurrent atrial fibrillation; therefore, a sensitivity analysis was performed excluding patients with preexisting atrial fibrillation and those who underwent concomitant arrhythmia surgery. As a secondary end point, major adverse cardiovascular and cerebrovascular events, defined as the composite of in-hospital all-cause mortality, stroke, and myocardial infarction, were evaluated.

### Statistical Analysis

Continuous variables are presented as means ± SDs or medians (interquartile ranges), as appropriate. Categorical variables are presented as numbers (percentages). Baseline characteristics were compared between the with sarcopenia and without sarcopenia groups using the chi-square or Fisher exact tests for categorical variables and unpaired Student *t* or Mann–Whitney *U* tests for continuous variables, as appropriate. The incidence of the primary outcome was compared between the groups using the chi-square test.

Logistic regression analyses were performed to evaluate which factors were associated with the primary outcome. In the overall cohort, univariate logistic regression analysis was conducted to assess the association between postoperative complications and the following variables: age, sex, sarcopenia, reduced ejection fraction (defined as left ventricular ejection fraction of <40%), prior stroke, chronic kidney disease, chronic obstructive pulmonary disease, and surgery type. Any variables deemed to be clinically relevant, age, sex, sarcopenia, reduced ejection fraction, and prior stroke history, were included in the multivariate logistic regression model. Sex-stratified univariate logistic regression analyses were also performed to evaluate the association between sarcopenia and the primary outcome separately in the male and female patients. A sensitivity analysis was then conducted excluding any patients with preexisting atrial fibrillation to assess the robustness of the association between sarcopenia and postoperative complications. All statistical analyses were performed using SPSS version 28 (IBM Corp) and R version 4.2.1 (R Foundation for Statistical Computing).

## Results

### Study Population

A total of 472 patients who underwent open surgeries at Nippon Medical School Hospital between July 2020 and August 2024 were initially screened. After excluding those who underwent nonelective surgeries (n = 114), those aged less than 65 years (n = 170), and those with insufficient clinical or imaging data (n = 55), 133 patients were included in the final analysis. Among them, 49 (36.8%) were classified as with sarcopenia, and 84 (63.2%) were classified as without sarcopenia (Figure 1).

**Figure 1:**
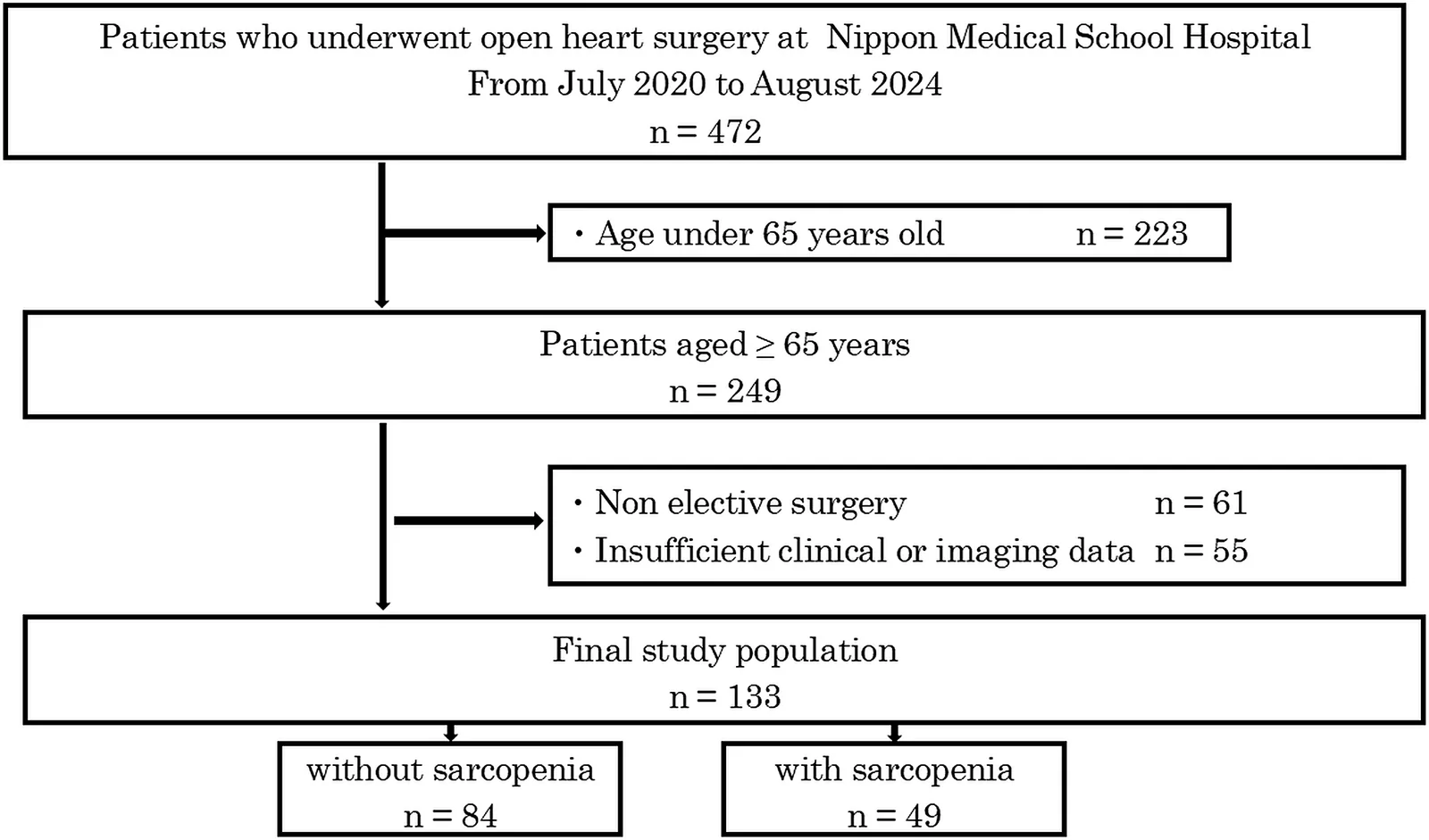
Flowchart of the patient selection process for this study. Of 472 patients who were initially screened, 133 met the inclusion criteria and were included in the final analysis, after the exclusion of those undergoing nonelective surgeries, those aged less than 65 years, and those with insufficient data. The final cohort comprised 49 patients with sarcopenia and 84 patients without sarcopenia.

### Baseline Characteristics and Incidence of Postoperative Complications

The baseline characteristics of the overall cohort are summarized in Tables 1 and 2. No significant differences were observed concerning major clinical characteristics between the patients with and without sarcopenia. In the overall cohort, the incidence of postoperative complications was numerically higher in the patients with sarcopenia versus those without sarcopenia; however, this difference did not reach statistical significance.

**Table 1:** Baseline characteristics of elective cardiac surgery in older patients with and without sarcopenia

Presenting characteristics	Overall cohort			Male			Female		
	Without sarcopenia	With sarcopenia	*P* value	Without sarcopenia	With sarcopenia	*P* value	Without sarcopenia	With sarcopenia	*P* value
N	84	49		59	37		25	12	
Age, y	73.0 [70.0-77.0]	73.0 [70.0-77.0]	.846	73.0 [70.0-77.0]	73.0 [70.0-77.0]	.850	73.0 [70.0-77.0]	75.0 [68.7-77.0]	1.000
Body mass index, kg/m^2^	22.9 [20.5-24.8]	22.7 [20.6-25.4]	.887	23.5 [21.6-25.3]	24.1 [21.1-26.4]	.964	21.8 [18.5-24.5]	20.9 [19.7-22.7]	.758
Alb, g/dL	4.2 [4.0-4.4]	4.2 [3.9-4.4]	.336	4.2 [4.0-4.4]	4.3 [3.8-4.5]	.919	4.3 [4.0-4.6]	4.1 [3.9-4.3]	.117
Hb, g/dL	13.2 [12.0-14.1]	13.1 [11.5-14.1]	.726	13.6 [12.6-14.3]	13.1 [12.4-14.5]	.580	12.8 [11.7-13.5]	12.9 [11.4-13.6]	.935
eGFR, mL/min/1.73 m^2^	54.5 [43.7-64.2]	54.0 [38.0-66.0]	.746	55.0 [45.0-64.0]	55.0 [39.0-66.0]	.857	54.0 [43.0-64.0]	51.0 [34.2-57.7]	.291
Medical history									
Hypertension	57 (68.7)	26 (53.1)	.108	40 (69.0)	23 (62.2)	.644	17 (68.0)	3 (25.0)	.035
Diabetes	26 (31.3)	18 (36.7)	.656	22 (37.9)	15 (40.5)	.969	4 (16.0)	3 (25.0)	.837
Chronic kidney disease	13 (15.7)	9 (18.4)	.872	11 (19.0)	6 (16.2)	.947	2 (8.0)	3 (25.0)	.367
Dialysis	2 (2.4)	2 (4.1)	.987	1 (1.7)	1 (2.7)	1.000	1 (4.0)	1 (8.3)	1.000
Pulmonary insufficiency	4 (4.8)	6 (12.2)	.224	4 (6.9)	5 (13.5)	.475	0 (0.0)	1 (8.3)	.704
Liver dysfunction	4 (4.8)	0 (0.0)	.301	3 (5.2)	0 (0.0)	.421	1 (4.0)	0 (0.0)	1.000
Cancer	14 (16.9)	10 (20.4)	.783	11 (19.0)	8 (21.6)	.958	3 (12.0)	2 (16.7)	1.000
Stroke	10 (12.0)	4 (8.2)	.683	9 (15.5)	3 (8.1)	.457	1 (4.0)	1 (8.3)	1.000
Coronary artery disease	35 (42.2)	19 (39.6)	.916	28 (48.3)	16 (43.2)	.788	7 (28.0)	3 (27.3)	1.000
Smoking, current or ex	47 (56.6)	30 (61.2)	.738	39 (67.2)	28 (75.7)	.517	8 (32.0)	2 (16.7)	.557
12-lead ECG			.775			.588			.306
Sinus rhythm	73 (88.0)	41 (83.7)		53 (91.4)	33 (89.2)				
Atrial fibrillation	9 (10.8)	7 (14.3)		4 (6.9)	4 (10.8)		5 (20.0)	3 (25.0)	
Pacemaker	1 (1.2)	1 (2.0)		1 (1.7)	0 (0.0)		0 (0.0)	1 (8.3)	
Echocardiography									
LVEF, %	65.0 [55.0-71.0]	63.0 [50.0-69.0]	.288	64.0 [53.0-68.0]	63.0 [49.0-69.0]	.879	68.0 [63.0-74.0]	64.0 [52.5-65.2]	.118
Spirometry									
FEV1%, %	74.3 [70.2-79.0]	73.6 [67.5-77.2]	.444	76.0 [70.6-79.7]	71.2 [65.8-76.4]	.036	72.2 [69.7-75.5]	76.4 [74.9-80.5]	.017
Computed tomography									
Right psoas muscle area, cm^2^	6.1 [4.9-7.6]	3.9 [2.7-4.7]	<.001	6.7 [5.8-8.6]	4.4 [3.6-4.7]	<.001	3.7 [3.1-5.2]	2.2 [1.5-2.8]	<.001
Left psoas muscle area, cm^2^	7.1 [5.3-8.8]	4.4 [3.3-5.7]	<.001	8.0 [6.9-9.5]	4.9 [3.9-5.8]	<.001	3.9 [3.4-4.7]	2.7 [2.4-3.4]	<.001
BSA, m^2^	1.6 [1.5-1.7]	1.5 [1.4-1.7]	.315	1.7 [1.6-1.8]	1.6 [1.5-1.8]	.197	1.4 [1.3-1.5]	1.4 [1.3-1.5]	.673
PAI	8.1 [7.0-9.4]	5.2 [4.2-6.0]	<.001	8.7 [7.8-10.6]	5.6 [4.8-6.2]	<.001	5.2 [4.8-6.8]	4.0 [2.7-4.2]	<.001
Medications									
β-blockers, n (%)	37 (44.6)	29 (59.2)	.150	30 (51.7)	23 (62.2)	.431	7 (28.0)	6 (50.0)	.345
ACEi, n (%)	4 (4.8)	6 (12.2)	.224	4 (6.9)	4 (10.8)	.771	0 (0.0)	2 (16.7)	.186
ARB, n (%)	34 (41.0)	15 (30.6)	.316	25 (43.1)	14 (37.8)	.768	9 (36.0)	1 (8.3)	.168
ARNI, n (%)	8 (9.6)	3 (6.1)	.704	6 (10.3)	2 (5.4)	.641	2 (8.0)	1 (8.3)	1.000
Diuretics, n (%)	32 (38.6)	25 (51.0)	.224	21 (36.2)	19 (51.4)	.213	11 (44.0)	6 (50.0)	1.000
Antiplatelet drugs, n (%)	32 (38.6)	16 (32.7)	.622	27 (46.6)	10 (27.0)	.092	5 (20.0)	6 (50.0)	.138
Antithrombotic drug, n (%)	17 (20.5)	12 (24.5)	.749	12 (20.7)	7 (18.9)	1.000	5 (20.0)	5 (41.7)	.320

Values are presented as mean ± SD. *ACEi*, Angiotensin-converting enzyme inhibitor; *Alb*, albumin; *ARB*, angiotensin II receptor blocker; *ARNI*, angiotensin receptor–neprilysin inhibitor; *BMI*, body mass index; *BSA*, body surface area; *CKD*, chronic kidney disease; *COPD*, chronic obstructive pulmonary disease; *ECG*, electrocardiogram; *eGFR*, estimated glomerular filtration rate; *FEV1*, forced expiratory volume in 1 second; *Hb*, hemoglobin; *LVEF*, left ventricular ejection fraction; *PAI*, psoas muscle area index.

**Table 2:** In-hospital composite complications of elective cardiac surgery in older patients with and without sarcopenia

Characteristics	Overall cohort			Male			Female		
	Without sarcopenia	With sarcopenia	*P* value	Without sarcopenia	With sarcopenia	*P* value	Without sarcopenia	With sarcopenia	*P* value
N	84	49		59	37		25	12	
In-hospital composite complications	32 (38.1)	26 (53.1)	.134	20 (33.9)	21 (56.8)	.046	12 (48.0)	5 (41.7)	.992
Stroke	1 (1.2)	1 (2.0)	1.000	0 (0.0)	1 (2.7)	.813	1 (4.0)	0 (0.0)	1.000
Renal failure	2 (2.4)	4 (8.2)	.264	1 (1.7)	3 (8.1)	.315	1 (4.0)	1 (8.3)	1.000
Reoperation for bleeding	2 (2.4)	1 (2.0)	1.000	1 (1.7)	1 (2.7)	1.000	1 (4.0)	0 (0.0)	1.000
Cardiac arrest	0 (0.0)	2 (4.1)	.260	0 (0.0)	2 (5.4)	.284			
Atrial fibrillation	29 (34.5)	20 (40.8)	.590	17 (28.8)	16 (43.2)	.219	12 (48.0)	4 (33.3)	.625
Prolonged ventilation	6 (7.1)	9 (18.4)	.091	4 (6.8)	6 (16.2)	.259	2 (8.0)	3 (25.0)	.367
Reintubation	2 (2.4)	5 (10.2)	.122	1 (1.7)	3 (8.1)	.315	1 (4.0)	2 (16.7)	.498
Tamponade	0 (0.0)	1 (2.0)	.784	0 (0.0)	1 (2.7)	.813			
Septicemia	1 (1.2)	2 (4.1)	.633	1 (1.7)	2 (5.4)	.679			
Gastrointestinal complication	0 (0.0)	1 (2.0)	.784	0 (0.0)	1 (2.7)	.813			
MACCE	1 (1.2)	2 (4.1)	.633	0 (0.0)	2 (5.4)	.284	1 (4.0)	0 (0.0)	1.000

*MACCE*, Major adverse cardiovascular and cerebrovascular events, defined as the composite of in-hospital all-cause death, stroke, and myocardial infarction.

The sex-stratified baseline characteristics and outcomes of the patients are presented in Tables 1 and 2. Among the male patients (n = 96), 37 (38.5%) were classified as having sarcopenia. These patients exhibited a significantly higher incidence of postoperative complications compared with their male counterparts without sarcopenia (56.8% vs 33.9%, *P* = .046). Conversely, among the female patients (n = 37), 12 (32.4%) were classified as having sarcopenia, and no significant difference in the incidence of composite complications was observed between the sarcopenia and nonsarcopenia groups (41.7% vs 48.0%, *P* = .992).

With respect to individual components, the composite end point did not differ significantly between the with sarcopenia and without sarcopenia groups in the overall cohort or the sex-stratified analyses. The incidence of major adverse cardiovascular and cerebrovascular events did not differ significantly between the sarcopenia and nonsarcopenia groups.

### Logistic Regression Analysis in the Overall Cohort

Univariate logistic regression analysis in the overall cohort demonstrated that sarcopenia was not significantly associated with the occurrence of postoperative complications (odds ratio, 1.83, 95% CI, 0.90-3.77). Other clinical variables, including age, sex, reduced ejection fraction, prior stroke, chronic kidney disease, chronic obstructive pulmonary disease, and type of surgery, were also not significantly associated with the primary outcome (Table 3).

**Table 3:** Logistic regression analysis for in-hospital composite complications

Characteristics	Univariate		Multivariate		Sensitivity analysis (AF excluded) multivariate	
	Odds ratio (95% CI)	*P* value	Adjusted odds ratio (95% CI)	*P* value	Adjusted odds ratio (95% CI)	*P* value
Sarcopenia	1.83 (0.90-3.77)	.095	1.98 (0.97-4.09)	.061	3.73 (1.49-9.84)	.006
Age	0.99 (0.92-1.07)	.974	0.99 (0.92-1.07)	.940	1.06 (0.96-1.17)	.231
Male	0.87 (0.40-1.89)	.736	0.78 (0.36-1.70)	.539	0.67 (0.25-1.88)	.436
rEF	0.93 (0.29-2.95)	.912	0.94 (0.37-2.33)	.900	1.15 (0.32-3.54)	.810
Stroke	1.34 (0.36-5.02)	.656	1.21 (0.38-3.70)	.737	1.47 (0.29-5.72)	.596
CKD	1.30 (0.15-11.13)	.794				
COPD	0.85 (0.11-5.34)	.868				
Type of surgery	1.30 (0.15-11.13)	.794				

*AF*, Atrial fibrillation; *CKD*, chronic kidney disease; *COPD*, chronic obstructive pulmonary disease; *rEF*, reduced ejection fraction (left ventricular ejection fraction <40%).

In the sensitivity analysis excluding patients with preexisting atrial fibrillation, multivariate logistic regression analysis revealed that sarcopenia was independently associated with a higher risk of postoperative complications (odds ratio, 3.73, 95% CI, 1.49-9.84, *P* = .006). No other clinical variables were significantly associated with the primary outcome in this model.

### Sex-Stratified Logistic Regression Analysis

Our sex-stratified logistic regression analyses are presented in Table 4. In the male patients, sarcopenia was significantly associated with the occurrence of postoperative complications in both univariate and multivariate logistic regression analyses after adjusting for the clinically relevant covariates. Conversely, no significant association between sarcopenia and the primary outcome was observed in the female patients through univariate or multivariate analyses.

**Table 4:** Sex-stratified logistic regression analyses for in-hospital composite complications

Characteristics	Male				Female			
	Univariate		Multivariate		Univariate		Multivariate	
	Odds ratio (95% CI)	*P* value	Adjusted odds ratio (95% CI)	*P* value	Odds ratio (95% CI)	*P* value	Adjusted odds ratio (95% CI)	*P* value
Sarcopenia	2.55 (1.10-6.05)	.029	2.52 (1.08-6.01)	.033	0.77 (0.18-3.09)	.718	0.72 (0.15-3.18)	.673
Age	1.00 (0.91-1.09)	.984	1.00 (0.91-1.10)	.929	0.99 (0.86-1.13)	.911	0.99 (0.86-1.13)	.920
rEF	1.65 (0.62-4.38)	.315	1.23 (0.43-3.44)	.684	2.09 (0.25-16.86)	.489	0.43 (0.02-3.95)	.494
Stroke	0.50 (0.12-1.98)	.324	0.71 (0.17-2.61)	.626	4.36 (0.35-53.38)	.249		
CKD	1.35 (0.05-34.83)	.834			1.18 (0.04-31.66)	.906		
COPD	0.88 (0.11-5.61)	.900						
Type of surgery	1.35 (0.05-34.83)	.834			1.18 (0.04-31.66)	.906		

*CKD*, Chronic kidney disease; *COPD*, chronic obstructive pulmonary disease; *rEF*, reduced ejection fraction (left ventricular ejection fraction <40%).

## Discussion

In this retrospective observational study of patients aged 65 years or more who underwent elective open surgeries, preoperative CT-defined sarcopenia was associated with an increased risk of postoperative complications in the male patients, but not in female patients. These findings highlight the importance of sex-specific assessments when evaluating the potential prognostic impacts of sarcopenia in older cardiac surgery candidates.

Previous studies have demonstrated that sarcopenia is associated with adverse outcomes after various types of cardiac surgeries, including coronary artery bypass grafting, valve surgery, and major aortic surgery.^14^, ^15^, ^16^, ^17^, ^18^ In particular, CT-based assessments of skeletal muscle mass using the PAI have been shown to predict both short- and long-term outcomes in older surgical patients.^16^, ^17^, ^18^, ^19^ However, most prior studies on this matter have evaluated sarcopenia in mixed populations, without detailed considerations of sex-specific differences.^21^, ^22^, ^23^ Our findings extend this existing evidence by demonstrating that the prognostic significance of CT-defined sarcopenia may differ substantially between male and female patients.

Several mechanisms may explain the sex-specific association between sarcopenia and postoperative complications that we observed. Men generally have greater absolute skeletal muscle mass and thus experience more pronounced age-related muscle loss than women.^24^ Therefore, muscle mass indicators measured by CT scanning may more accurately reflect physiological reserve capacity and vulnerability to surgical stress in male patients. Women, conversely, tend to have lower basal muscle mass and different body composition patterns, which may limit the applicability of current CT-based cutoff values.^24^ These differences suggest that current sarcopenia definitions based solely on muscle mass may inadequately capture frailty-related risks in female patients undergoing cardiac surgeries.^25^

The prognostic impact of sarcopenia may be diluted if sex-based differences remain unaccounted for, which may explain the inconsistent findings reported in previous studies.^21^ Our sensitivity analysis excluding patients with preexisting atrial fibrillation further supports the robustness of the association between sarcopenia and postoperative complications in selected patient subgroups.^26^ Notably, no significant differences were observed in the individual components of the composite end point between our with sarcopenia and without sarcopenia patient groups. This finding suggests that sarcopenia may contribute to a generalized vulnerability to postoperative complications, rather than being associated with specific adverse events. Sarcopenia likely reflects reduced physiological reserve, impaired stress response, and limited capacity for postoperative recovery. Collectively, these features may increase patient susceptibility to various complications during hospitalization.^27^^,^^28^

Our findings have several important clinical implications. First, CT-based assessment of sarcopenia using routinely obtained preoperative imaging may serve as a practical tool for perioperative risk stratification, particularly in male patients. Unlike functional measures, CT-based assessment facilitates the objective quantification of muscle mass, even in acute settings where conventional physical performance tests are rarely feasible. Second, sex-specific approaches may be necessary when applying sarcopenia-related risk models in cardiac surgeries. Third, we performed the sensitivity analysis because preexisting atrial fibrillation is a well-established risk factor for postoperative complications^26^ and may be associated with sarcopenia through shared mechanisms such as inflammation and frailty. This sensitivity analysis was hypothesis-driven, given the dual role of atrial fibrillation as both a component of the composite end point and a potential confounder. Excluding these patients allowed us to confirm that the observed association was not solely driven by atrial fibrillation–related risk. Finally, identifying sarcopenia preoperatively may provide an opportunity to implement targeted interventions such as prehabilitation, nutritional optimization, and early postoperative rehabilitation to mitigate postoperative risk.

### Limitations

This study had some limitations. First, its retrospective, single-center design may limit the generalizability of our findings. Second, sarcopenia was defined solely by CT-derived muscle mass, without any assessment of muscle strength or physical performance. This may have led to some misclassification, particularly in the female patients. Third, most events were driven by atrial fibrillation, whereas the number of non–atrial fibrillation events was limited, restricting the ability to detect differences in individual complications. In this context, exclusion of patients with preexisting atrial fibrillation reduced the at-risk population, whereas the number of postoperative events, predominantly new-onset atrial fibrillation, remained largely unchanged. Thus, the observed association should be interpreted primarily as reflecting the risk of postoperative atrial fibrillation rather than overall complications. Fourth, the sample size was relatively small, particularly in the female subgroup, which may have limited the statistical power to detect associations. In addition, baseline imbalances, such as differences in hypertension prevalence and pulmonary function parameters among female patients, were observed and may be due to the limited sample size. Among female patients, postoperative atrial fibrillation occurred more frequently in the nonsarcopenia group, which may have contributed to the higher composite complication rate; however, the small number of female patients precluded definitive interpretation. The limited sample size also reduced the statistical power to detect significant differences in individual postoperative complications and in sex-specific subgroup analyses, particularly among women. Future multicenter, multinational prospective studies including patients with diverse ethnic backgrounds and body sizes are warranted to validate the generalizability of CT-defined sarcopenia as an objective marker of frailty. In addition, selection bias may have influenced the findings. To evaluate this possibility, patients aged 65 years or more who were excluded from the analysis were compared with the study cohort (Table E1). The excluded cohort, which included patients who underwent nonelective surgery, had poorer clinical status and higher in-hospital mortality than the study cohort. Therefore, caution is warranted when generalizing these findings to higher-risk populations.

## Conclusions

Preoperative CT-defined sarcopenia was independently associated with postoperative complications in a cohort of male Japanese patients aged 65 years or more who underwent elective open surgeries, but not in their female counterparts. These findings suggest that the prognostic significance of sarcopenia in cardiac surgeries is sex specific and that current muscle mass–based definitions may be more applicable to men. Further large-scale prospective studies are warranted to refine sex-specific risk stratification strategies in older candidates for cardiac surgery.

## Conflict of Interest Statement

The authors reported no conflicts of interest.

The *Journal* policy requires editors and reviewers to disclose conflicts of interest and to decline handling or reviewing manuscripts for which they may have a conflict of interest. The editors and reviewers of this article have no conflicts of interest.

## Figures and Tables

**Table E1:** Comparison of baseline characteristics between the inclusion and exclusion cohorts

	Overall cohort		
	Inclusion cohort	Exclusion cohort	*P* value
N	133	116	
Age, y	73.0 [70.0-77.0]	74.0 [70.0-77.0]	.181
Male, n (%)	96 (72.2)	68 (58.6)	.032
Body mass index, kg/m^2^	22.9 [20.5-24.8]	23.0 [20.8-25.7]	.938
Alb, g/dL	4.2 [4.0-4.4]	3.6 [3.0-4.0]	<.001
Hb, g/dL	13.2 [12.0-14.1]	12.2 [10.2-13.6]	<.001
eGFR, mL/min/1.73 m^2^	54.5 [43.7-64.2]	48.0 [34.0-64.0]	.067
β-blockers, n (%)	37 (44.6)	29 (25.0)	<.001
Diuretics, n (%)	32 (38.6)	26 (22.4)	<.001
Antiplatelet drugs, n (%)	32 (38.6)	13 (11.2)	<.001
Antithrombotic drug, n (%)	17 (20.5)	7 (6.0)	<.001
In-hospital all-cause death, n (%)	0 (0)	13 (11.2)	<.001

Values are presented as mean ± SD. *Alb*, Albumin; *Hb*, hemoglobin; *eGFR*, estimated glomerular filtration rate.
